# Effect of PEG on Performance of NiMnO Catalyst for Hydrogen Evolution Reaction

**DOI:** 10.3389/fchem.2020.00281

**Published:** 2020-04-23

**Authors:** Jinfeng Zeng, Lu Chen, Linlin Li, Wei Yang, Hanbo Zou, Shengzhou Chen

**Affiliations:** ^1^School of Chemistry and Chemical Engineering, Guangzhou University, Guangzhou, China; ^2^School of Biotechnology and Health Science, Wuyi University, Jiangmen, China; ^3^Guangzhou Key Laboratory for New Energy and Green Catalysis, Guangzhou University, Guangzhou, China

**Keywords:** hydrogen evolution reaction, electrocatalysts, nickel-manganese oxides, surfactant, polyethylene glycol

## Abstract

Solvothermal method is a very common synthetic method in the preparation of catalysts for the hydrogen evolution reaction (HER) of H_2_O decomposition. Since a certain surfactant can be added to the solvothermal solvent, the crystal particle growth process can be changed to obtain catalysts with different morphologies. We synthesized a series of nickel-manganese oxides (NiMnO) by adding different amounts of Polyethylene glycol (PEG) using the solvothermal method. Structure characterizations exhibit that NiMnO catalyst prepared with different PEG additions have different morphologies. The NiMnO catalyst prepared by adding 3 g of PEG possesses abundant petal-like scales, it brings a large specific surface area, high reaction efficiency, and has the best electrocatalytic activity in alkaline media.

## Introduction

The world's energy consumption of fossil energy such as coal, oil, and natural gas brings many questions, such as energy shortage and serious environmental pollution (Zou and Zhang, 2015; Ojha et al., 2018). H_2_ is a promising clean and renewable source of energy with extremely high calorific value (285.8 kJ mol^−1^). Electrocatalytic cracking water to H_2_ becomes feasible for the impetus of electrochemical HER is derived from electricity from tidal energy, wind energy, geothermal energy, etc. Researchers have found that platinum-based catalysts are the most efficient catalysts for HER (Chen et al., 2013; Zhou W. et al., 2016; Wu et al., 2019; Eiler et al., 2020; Li and Baek, 2020). However, platinum is very rare in the earth's crust, making it extremely expensive. Therefore, it is necessary to develop a metal catalyst with abundant resources as an alternative. After the efforts of scientists, they found that transition metal oxide is an efficient catalyst for the HER of H_2_O decomposition (Wang et al., 2017; Han et al., 2019; Liang et al., 2020).

The atomic number of nickel is 28. The abundance of nickel in Earth's crust is > 50 ppm. And nickel is an earth-abundant first row transitional metal (Jamesh et al., 2015). Ni possesses very strong ductility and corrosion-resistance. The addition of nickel could significantly improve the stability and activity of catalyst for HER (Chu et al., 2018; Zhang et al., 2019). Another rich transition metal element manganese on the earth is also an important catalyst raw material. There are many defects, which created in MnO_2_, can strengthen the stability and activity for HER. δ-MnO_2_ Nanosheet demonstrates favorable stability and activity for HER (Leonard and Bard, 2013; Liu et al., 2017; Zhao et al., 2017). The δ-MnO_2_ phase includes trivalent and tetravalent manganese. It will create defects in the catalyst structure. Therefore, the defects and oxygen vacancies can generate the Mn^3+^ active sites, which can afford half-metalicity. This can significantly increase the electronic conductivity of the catalyst, and thus the reaction efficiency is significantly improved (Mohammed-Ibrahim and Sun, 2019).

The performance of HER catalysts is highly correlated to the synthetic methods. On account of the inherent pitfalls, i.e., unable to control the concentration gradient and dispersibility of metal particles (Arevalo and Chirik, 2019), the traditional synthetic methods, such as precipitation and wet impregnation, have been gradually phased out. For the past few years, by combining surfactant with traditional co-precipitation method was raised and widespread used for catalyst preparation (Das et al., 2015; Chong et al., 2018; Xu et al., 2018; Toloman et al., 2019).

Due to the hydroxyl protons exchange between cetyltrimethyl ammonium bromide (CTAB) and hydrous metal hydroxide gels would lower surface tension and interfacial energy of H_2_O exist in the pores (Sohn and Ozkan, 2016). Therefore, CTAB can assist-synthesized a high specific surface area oxide catalyst. Moreover, the pore structure did not collapse during the calcination process. Likewise, polyvinyl pyrrolidone (PVP), a non-ionic polymer surfactant could play a similar role (Tan et al., 2014; Wang et al., 2019). In addition, some surfactants possess amphiphilic property, This series of molecules can generate thermodynamically stable gathers of intrinsically nano-scale both in solution and at the solid liquid interphases (Lee et al., 2019; Liu et al., 2019). Surfactants have a unique ability to self-organize at interface or in solution, modify interfacial properties and enhance the compatibility between materials of very different characteristics (Carswell et al., 2003; Hosseini et al., 2016).

In this article, NiMnO catalysts were synthesized by polyethylene glycol surfactant assist- solvent-thermal method, and then the electrochemical catalytic properties for HER were estimated. The as-prepared catalysts were explored by N_2_ adsorption-desorption, X-ray diffraction (XRD), and scanning electron microscopy (SEM). Besides, we have performed a series of electrochemical tests (cyclic voltammetry, linear sweep voltammetry, electrochemical impedance spectroscopy, and chronopotentiometry), and derived some electrochemical parameters (Tafel slopes, electrochemical double-layer capacitance, and Turnover frequency).

## Experimental

### Sample Synthesis

Materials: Polyethylene glycol (PEG, M_w_ = 20,000) and ethanol were supplied by Guangzhou Chemical Reagent Factory. Ni(CH_3_COO)_2_•4H_2_O, Mn(CH_3_COO)_2_•4H_2_O and urea were supplied by Shanghai Macklin Biochemical Co., Ltd. The water (18.25 mΩ cm^−1^) used in the experiments was purified by Millipore system (Milli-Q Academic). All reagents used throughout this manuscript were analytical reagent grade.

Synthesis of NiMnO catalyst: Typically, 2.043 g of Ni(CH_3_COO)_2_•4H_2_O and 3.036 g of Mn(CH_3_COO)_2_•4H_2_O were placed in 40 mL anhydrous ethanol to form a homogeneous solution under magnetic stirring for 2 h at room temperature, 7 g of Urea was dissolved in 10 mL deionized water, different weight of PEG (0, 2, and 3 g) were dispersed in 10 mL anhydrous ethanol under magnetic stirring for 1 h at room temperature, which resulting in three samples, marked as NMO, NMO-PEG-2g, and NMO-PEG-3g. Then, The PEG suspension and the Urea solution were dripped into the Ni(CH_3_COO)_2_•4H_2_O-Mn(CH_3_COO)_2_•4H_2_O solution in sequence under stirring. Kept stirring for 2 h at room temperature, the mixture was transferred into the Teflon-lined stainless-steel autoclave and heat at 180°C for 10 h for the solvent-thermal reaction. After the reaction was completed and being cooled to room temperature, the precipitates were collected by centrifugation and washed with deionized water and ethanol for three times, then dried in an oven at 80°C for 6 h to get catalyst. After dried, the catalyst was subjected to bake in a muffle furnace at 750°C for 120 min.

### Microstructural Characterizations

X-ray diffraction (XRD) data was obtained by a PANalytical PW3040/60 X-ray diffractometer with Cu *K*α radiation (40 kV, 30 mA). Scanning electron microscope (SEM) measurements were observed by a ZEISS-Merlin microscope at 10 kV. Brunauer-Emmett-Teller (BET) surface area analysis (N_2_ adsorption) of the catalysts was obtained using an ASAP2020M system (Micromeritics). Inductively coupled plasma-atomic emission spectrometry (ICP-AES) was conducted on a PerkinElmer NexION 300X.

### Electrochemical Measurements

Five milligram NiMnO catalyst was dispersed in 10 mL isopropanol (Sigma-Aldrich) aqueous solution containing Nafion (15 wt.%, Sigma-Aldrich) (isopropanol: Nafion solution: water = 1:4:10 volume ratio). And then, sonicated for 30 min to formulate a homogeneous suspension (catalyst contain 0.5 mg mL^−1^). 10 μL of the above-mentioned suspension was coated on the glassy carbon electrode (CHI) with a diameter of 4 mm used as working electrode after infrared drying. Saturated calomel electrode (SCE) was used as reference electrode. Graphite flake was used as counter electrode. The electrolyte was 1.0 M KOH which was bubbled by high-purity argon 5 min before the measurement and continue until the measurement was completed. Cyclic voltammetry was conducted at 10 mV s^−1^. Linear sweep voltammetry (LSV) was collected by the glassy carbon electrode at a scan rate of 5mV s^−1^. Tafel slopes were calculated based on the corresponding LSV curves. Electrochemical impedance spectroscopy (EIS) was measured in the frequency scope from 105 to 0.01 Hz. The stability measurements were detected by chronopotentiometry at a constant current density of 10 mA mg^−2^. All voltages involved in the article vs. SCE were adjust to the reversible hydrogen electrode (RHE). Calibration using the following formula: E(RHE) = E(SCE) + 0.0591pH + 0.2415 – 0.000761 (*T* – 298.15). *T* represents the room temperature.

## Results and Discussion

The NiMnO catalyst was synthesized by a solvent-thermal route using urea as a precipitant. The chemical reactions involve the thermal decomposition of urea, following by the generation of ${\text{CO}}_{3}^{2-}$ ions and the subsequent precipitation of the NiMnO catalyst under solvothermal conditions.

$$\begin{array}{l} \text{N}{\text{H}}_{2}\text{CON}{\text{H}}_{2}+{\text{H}}_{2}\text{O}\to 2\text{N}{\text{H}}_{3}+\text{C}{\text{O}}_{2} \end{array}$$

$$\begin{array}{l} {\text{NH}}_{3}+{\text{H}}_{2}\text{O}\to {\text{NH}}_{4}^{+}+{\text{HO}}^{-} \end{array}$$

$$\begin{array}{l} {\text{CO}}_{2}+2{\text{OH}}^{-}\to {\text{CO}}_{3}^{2-}+{\text{H}}_{2}\text{O} \end{array}$$

$$\begin{array}{l} {\text{Ni}}^{2+}+{\text{Mn}}^{2+}+{\text{CO}}_{3}^{2-}\to {\text{NiMnCO}}_{3} \end{array}$$

**Figure 1:**
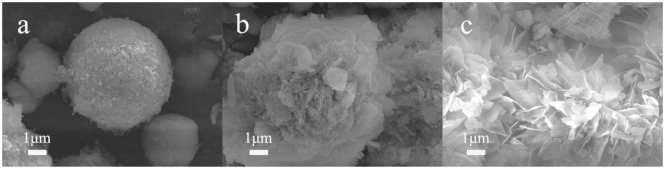
SEM images of **(a)** NMO, **(b)** NMO-PEG-2g, **(c)** NMO-PEG-3g.

**Figure 2:**
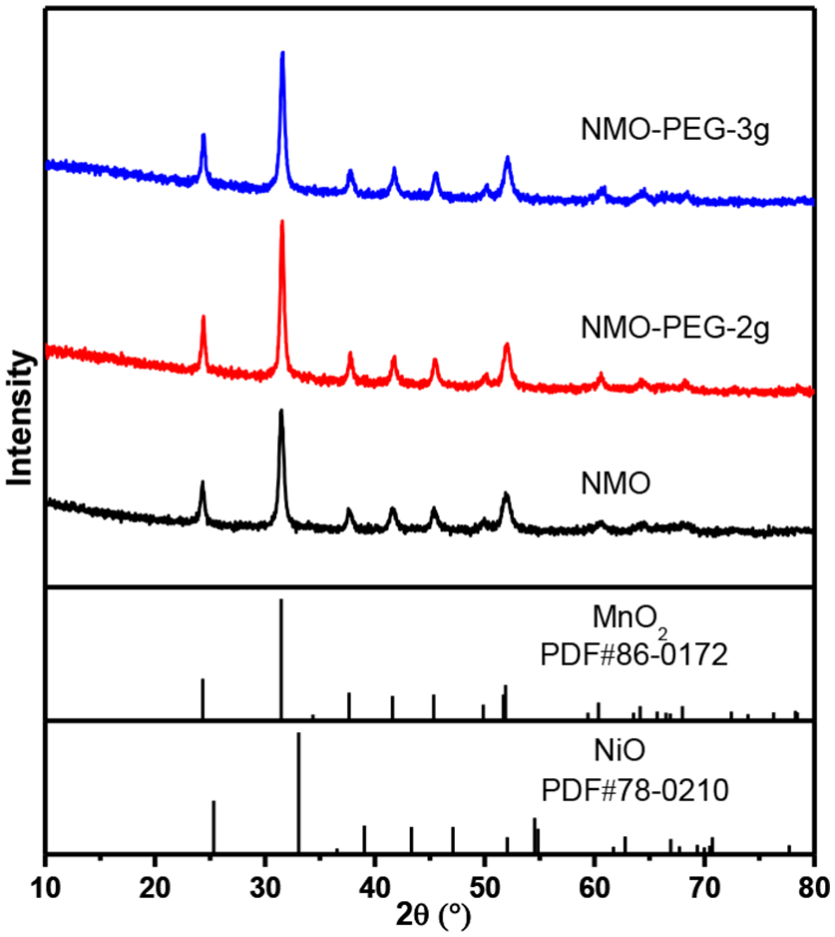
XRD patterns of NiMnO catalysts.

SEM images (Figure 1) show that the NiMnO can be successfully synthesized through solvent-thermal reaction. Different amounts of PEG resulted in different morphologies of the catalysts, among which the surface area of NMO-PEG-3g appeared to be largest. PEG can prevent NiMnCO_3_ from aggregation and compression. Meanwhile, the PEG with more mass represents more molecular chains, making it easier for PEG molecules to be entangled or spiraled with other PEG molecules through hydrogen bonds. This distortion will greatly increase the specific surface area of NMO due to the volatilization of PEG and CO_2_ after roasting with NMCO_3_ at 750°C. Larger specific surface area can increase the reaction active sites, which is beneficial to the catalytic activity. The N_2_ adsorption–desorption isotherms and pore-size distribution indicate that the NiMnO catalysts added PEG form a macropore structure. There are only a few micropores in the structure of the NiMnO catalyst without adding PEG (Figure 2 and Figure S2). The NiMnO produced when no PEG was added exhibited a 4–7 μm spherical structure. With the addition of PEG, scales appeared on the surface of the prepared catalyst. A well petal-like sheet has been formed in NMO-PEG-3g. And there are rich fluffy structures on the sheet. These structures are conducive to the electrochemical catalytic HER.

The composition and morphology of the electroactive catalysts were further investigated using XRD (Figure 2). The XRD peaks were indexed to crystal facets of MnO_2_ (JCPDS Card No. 86-0172) and NiO (JCPDS Card No. 78-0210). MnO_2_ has a smaller *K*_*sp*_ value than NiO, and [Mn(NH^3^)_n_]^2+^ has a smaller *K*_*f*_ value than [Ni(NH^3^)_n_]^2+^, which indicates that MnO_2_ is more easily precipitated than NiO (van Bommel and Dahn, 2009). Both MnO_2_ and NiO can be indexed to a typical hexagonal carbonate structure with a space group of *RC*. The accurate content of each element in NiMnO is further corroborated by SEM coupled energy-dispersive X-ray spectroscopy (EDS) (Figure S1) and ICP-AES (Table S1). The Ni and Mn contents in the product are calculated to be 8:92 (atomic ratio) from the EDS, which are in good agreement with the ICP-AES results.

**Figure 3:**
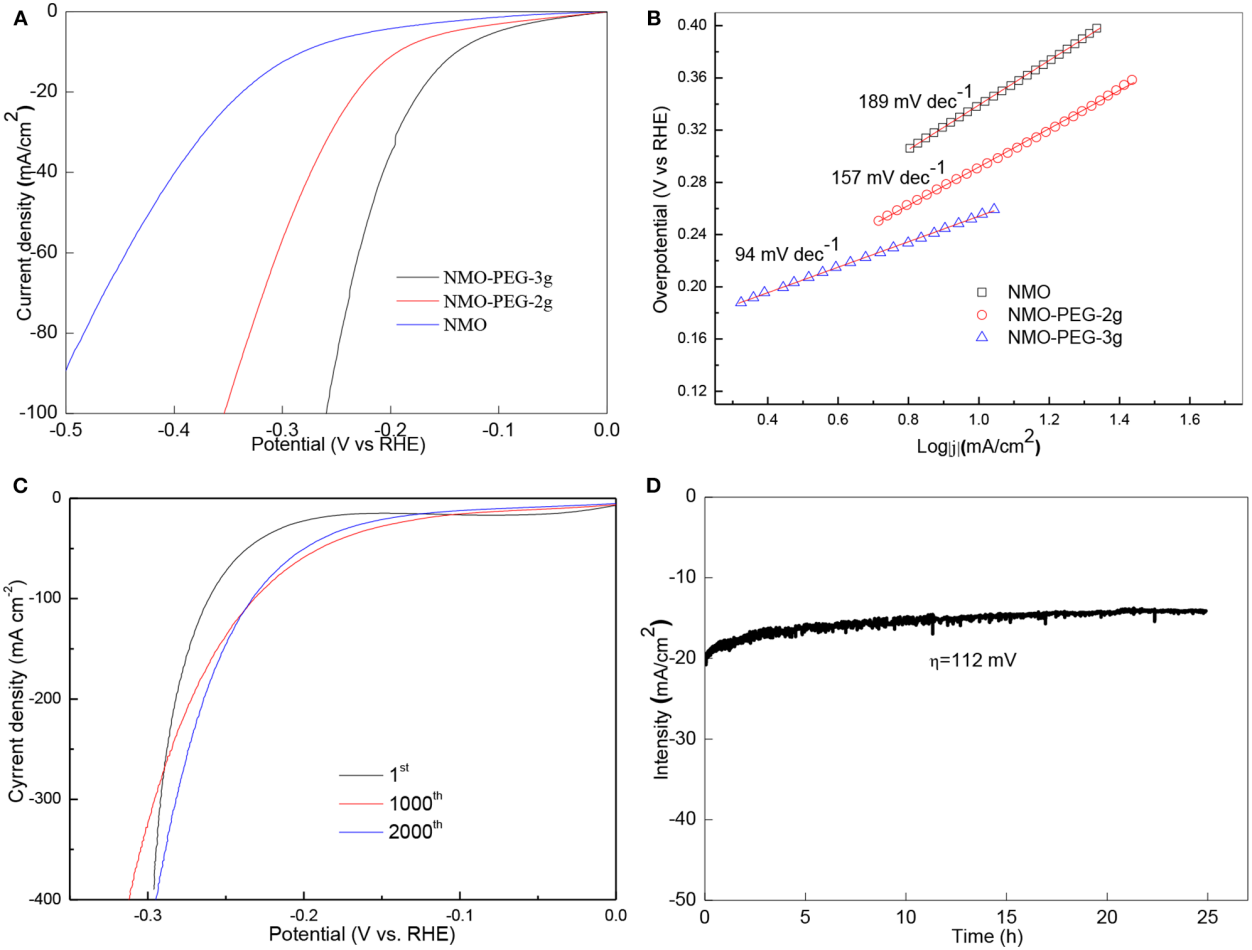
**(A)** LSV curves of NMO, NMO-PEG-2g, and NMO-PEG-3g detected in 1 M KOH at a sweep rate of 5 mV s^−1^; **(B)** Tafel plots for LSV curves of NMO, NMO-PEG-2g, and NMO-PEG-3g; **(C)** 1st, 1,000th, and 2,000th LSV curves at sweep rate of 50 mV s^−1^; **(D)** Stability test of NMO-PEG-3g catalyst at an over-potential of 112 mV for 25 h.

The electrocatalytic properties of NiMnO catalyst for HER in alkaline solution was evaluated using a standard three-electrode system. Comparing the three catalysts of NMO, NMO-PEG-2g, and NMO-PEG-3g, LSV results show that NMO-PEG-3g possesses excellent activities that are much better than the activity of NMO and NMO-PEG-2g (Figure 3A). The infrared- calibration polarization curve of NMO-PEG-3g exhibits favorable catalytic activity for HER with an onset potential of 36.3 mV. Under the same over-potential 0.1 V, NMO-PEG-3g exhibits better catalytic activity than that of NMO and NMO-PEG-2g. At 10 and 100 mA cm^−2^, the NMO-PEG-3g catalyst obtains 140 and 256 mV over-potential, respectively.

Tafel slope is an important property inherent of the HER catalyst. Figure 3B exhibits the Tafel plots of these catalysts' series. Accordingly, NMO-PEG-3g exhibits a small Tafel slope of 94 mV dec^−1^ (Figure 3B), which is much lower compared with NMO-PEG-2g (157 mV dec^−1^) and NMO (189 mV dec^−1^), demonstrating a faster HER kinetics of NMO-PEG-3g. These values mean the HER that occurred on NMO-PEG-3g catalyst follows the Volmer-Heyrovsky mechanism. The electrochemical desorption is the rate determining step. Stability is a fatal parameter that estimates the property of HER catalyst. At the LSV of 2,000th cycles, the performance of the NMO-PEG-3g improves than that at 1,000th cycles (Figure 3C). The cause of this phenomenon may be that the catalyst surface is activated in the process of electrochemical reaction, which fortifies reaction active sites. Figure 3D displays that NMO-PEG-3g endures the stability measurement of 112 mV for 25 h. As shown in Figure 3D, NMO-PEG-3g could keep a favorable HER current density at 14 mA cm^−2^.

The principal parameters of catalytic activities could be the amount of reactive sites, electrochemically active surface area (ECSA) and charge transfer rate (Qu et al., 2016; Du et al., 2017). The ECSA is crucial to the HER catalytic reaction procedure. The electrochemical double-layer capacitance (C_dl_) can be conducted to calculate the ECSA.

(1) $$\begin{array}{l} \text{ECSA}=\frac{C_{\text{dl}}}{C_{\text{s}}} \end{array}$$

**Figure 4:**
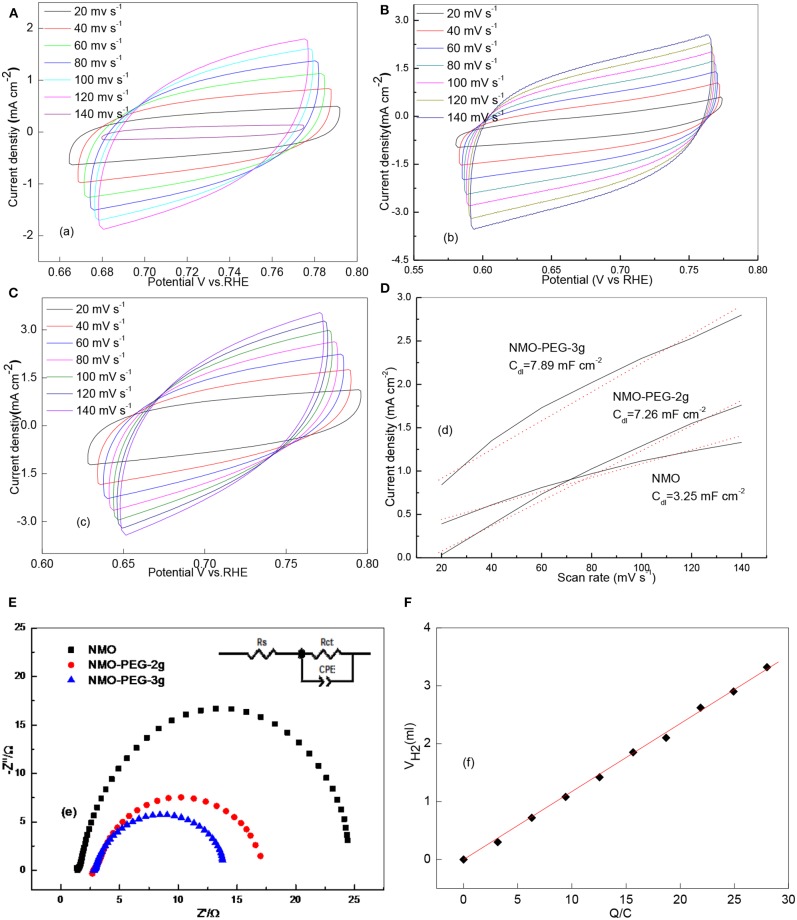
**(A)** CV curves of NMO; **(B)** CV curves of NMO-PEG-2g; **(C)** CV curves of NMO-PEG-3g (at different scan rates of 20, 40, 60, 80, 100, 120, and 140 mV s^−1^ in the non-faradaic potential region in 1 M KOH); **(D)** current density variation plotted against scan rate fitted to a linear regression enables the estimation of C_dl_; **(E)** Nyquist plots of as-prepared catalysts; **(F)** quantitative H_2_ measurement via water displacement.

C_s_ is the specific capacitance of the catalyst (mF cm^−2^). The Cs is generally valued as 0.040 mF cm^−2^ in 1 M KOH. Cyclic voltammetry test is proceeded to verify the C_dl_ at different scan rates in the non-faradaic potential region. As the chart shows, these test outcomes show that the as prepared NMO-PEG-3g possesses the biger C_dl_ than NMO and NMO-PEG-2g (Figures 4A–D). The C_dl_ of NMO-PEG-3g is derived to be 78.93 mF cm^−2^. The big numerical value of C_dl_ guarantees a competitive ECSA and efficient HER (shown in Table 1). BET specific surface area is identified to be immediate real surface area of catalyst (Sun et al., 2018). The S_BET_ of NMO-PEG-3g is 29.27 m^2^ g^−1^, which is bigger than those of NMO and NMO-PEG-2g.

**Table 1:** Electrochemical parameters of the NiMnO as the HER catalyst in alkaline medium.

**Electrodes**	**S_**BET**_ (m^**2**^ g^**−1**^)**	**ESCA (cm^**2**^)**	**TOF (s^**−1**^)**	**Rs (Ωcm^**2**^)**	**Rct (Ωcm^**2**^)**	**CPE (Fs^**n−1**^cm^**−2**^)**
NMO	2.16	81.25	0.067	1.1	23.2	0.0027
NMO-PEG-2g	22.35	181.5	0.041	2.5	14.4	0.0031
NMO-PEG-3g	29.27	197.25	0.035	2.53	10.6	0.0037

Turnover frequency (TOF) is a pivotal parameter to assess the HER activity of catalysts. TOF denotes the number of H_2_ molecules generated *per second* per active site. TOF was inspected according to a method reported by scientists (Popczun et al., 2013, 2014; Zhou H. et al., 2016). Thus, the number of electrochemically effective surface sites on the catalyst was calculated as the following Equations (2) and (3):

(2) $$\begin{array}{l} \;\frac{\#\;Surface\;sites\;\left(catalyst\right)}{cm^{2}\;geometric\;area}=\frac{\#\;Surface\;sites\;\left(flat\;standard\right)}{cm^{2}\;geometric\;area}\\ \times Roughness\;factor \end{array}$$

(3) $$\begin{array}{l} TOF\;per\;site=\frac{\frac{\#Total\;Hydrogen\;Turn\;Overs}{cm^{2}\;geometric\;area}}{\frac{\#Surface\;active\;sites}{cm^{2}geometric\;area}} \end{array}$$

Then, deductions reveal that NMO-PEG-3g catalyst has a faster TOF value compared to that of NMO and NMO-PEG-2g. This also proves why the catalyst possesses the favorable activity for electro-catalytic HER. The electrode kinetics for HER was further measured by EIS. EIS measurements and corresponding equivalent circuit in Figure 4E are collected to verify the catalytic reaction mechanism of as prepared catalysts for HER in alkaline media. NMO-PEG-3g exhibits a nice semicircle in the low frequency region. This proves a lower interfacial charge-transfer resistance (R_ct_ = 10.6 Ω) of NMO-PEG-3g than that of NMO and NMO-PEG-2g. This would be related to the increase in the number of surface-active sites and the reduced ΔG_M−H_ during the H^+^ discharge reaction step, resulting in superior HER kinetics. The petal-like scales in NMO-PEG-3g catalyst could provide a large reaction area, resulting in faster electron transfer rates and favorable kinetics for HER. As shown in Figure 4F, the quantitative H_2_ measurement demonstrates that the Faradic efficiency is close to 100%, which testifies that all charge generated in HER reaction procedures (Popczun et al., 2013) would be devoted to produce H_2_.

## Conclusions

In conclusion, the addition of PEG surfactant can directly affect the morphology of the prepared NiMnO catalyst by the solvothermal method. As prepared catalysts possess catalytic activities for the HER in alkaline media. Because the NiMnO catalyst prepared by adding 3 g of PEG possesses abundant petal-like scales, it brings a large specific surface area, high reaction efficiency, and has the best electrocatalytic activity. This experiment investigates that the morphology of the catalyst was controlled by changing the amount of surfactant to improve the catalytic activity of catalyst. Moreover, we will investigate the effect of PEG molecular weight on the structure of the prepared catalyst in subsequent experiments.

## Data Availability Statement

All datasets generated for this study are included in the article/Supplementary Material.

## Author Contributions

JZ, LC, and LL contributed conception and design of the study. JZ organized the database. LC performed the statistical analysis. LL wrote the first draft of the manuscript. WY, HZ, and SC wrote sections of the manuscript. All authors contributed to manuscript revision, read, and approved the submitted version.

## Conflict of Interest

The authors declare that the research was conducted in the absence of any commercial or financial relationships that could be construed as a potential conflict of interest.

## References

[B1] Arevalo R. Chirik P. J. (2019). Enabling two-electron pathways with iron and cobalt: from ligand design to catalytic applications. J. Am. Chem. Soc. 141, 9106–9123. 10.1021/jacs.9b03337 31084022 PMC6561843

[B2] Carswell A. D. W. O'Rea E. A. Grady B. P. (2003). Adsorbed surfactants as templates for the synthesis of morphologically controlled polyaniline and polypyrrole nanostructures on flat surfaces: from spheres to wires to flat films. J. Am. Chem. Soc. 125, 14793–14800. 10.1021/ja0365983 14640654

[B3] Chen W.-F. Muckerman J. T. Fujita E. (2013). Recent developments in transition metal carbides and nitrides as hydrogen evolution electrocatalysts. Chem. Commun. 49, 8896–8909. 10.1039/c3cc44076a 23982806

[B4] Chong C. C. Owgi A. H. K. Ainirazali N. Chin S. Y. Setiabudi H. D. (2018). CO_2_ reforming of CH_4_ over Ni/SBA-15 prepared by surfactant-assisted impregnation method: Comparative study of surfactant types. Mater. Today Proc. 5(10 Part 2), 21644–21651. 10.1016/j.matpr.2018.07.014

[B5] Chu M. Wang L. Li X. Hou M. Li N. Dong Y. (2018). Carbon coated nickel - nickel oxide composites as a highly efficient catalyst for hydrogen evolution reaction in acid medium. Electrochim. Acta 264, 284–291. 10.1016/j.electacta.2018.01.140

[B6] Das D. Llorca J. Dominguez M. Colussi S. Trovarelli A. Gayen A. (2015). Methanol steam reforming behavior of copper impregnated over CeO_2_-ZrO_2_ derived from a surfactant assisted coprecipitation route. Int. J. Hydrog. Energ. 40, 10463–10479. 10.1016/j.ijhydene.2015.06.130

[B7] Du K. Zheng L. Wang T. Zhuo J. Zhu Z. Shao Y.. (2017). Electrodeposited Mo_3_S_13_ films from (NH4)2Mo_3_S_13_·2H_2_O for electrocatalysis of hydrogen evolution reaction. ACS Appl. Mater. Interfaces 9, 18675–18681. 10.1021/acsami.7b01333 28524651

[B8] Eiler K. Suriñach S. Sort J. Pellicer E. (2020). Mesoporous Ni-rich Ni–Pt thin films: electrodeposition, characterization and performance toward hydrogen evolution reaction in acidic media. Appl. Catal. B Environ. 265:118597 10.1016/j.apcatb.2020.118597

[B9] Han C. Wang D. Li Q. Xing Z. Yang X. (2019). Ni_17_W_3_ nanoparticles decorated WO_2_ nanohybrid electrocatalyst for highly efficient hydrogen evolution reaction. ACS Appl. Energy Mater. 2, 2409–2413. 10.1021/acsaem.9b00170

[B10] Hosseini S. R. Ghasemi S. Ghasemi S. A. (2016). Effect of surfactants on electrocatalytic performance of copper nanoparticles for hydrogen evolution reaction. J. Mol. Liq. 222, 1068–1075. 10.1016/j.molliq.2016.08.013

[B11] Jamesh M. I. Li P. Bilek M. M. M. Boxman R. L. McKenzie D. R. Chu P. K. (2015). Evaluation of corrosion resistance and cytocompatibility of graded metal carbon film on Ti and NiTi prepared by hybrid cathodic arc/glow discharge plasma-assisted chemical vapor deposition. Corros. Sci. 97, 126–138. 10.1016/j.corsci.2015.04.022

[B12] Lee G.-J. Chen H.-C. Wu J. J. (2019). Enhancing the photocatalytic hydrogen evolution of copper doped zinc sulfide nanoballs through surfactants modification. Int. J. Hydrog. Energ. 44, 30563–30573. 10.1016/j.ijhydene.2018.02.041

[B13] Leonard K. C. Bard A. J. (2013). The study of multireactional electrochemical interfaces via a tip generation/substrate collection mode of scanning electrochemical microscopy: the hydrogen evolution reaction for Mn in acidic solution. J. Am. Chem. Soc. 135, 15890–15896. 10.1021/ja407395m 24063768

[B14] Li C. Baek J.-B. (2020). Recent advances in noble metal (Pt, Ru, and Ir)-based electrocatalysts for efficient hydrogen evolution reaction. ACS Omega 5, 31–40. 10.1021/acsomega.9b03550 31956748 PMC6963895

[B15] Liang Y. Yang Y. Xu K. Yu T. Yao S. Peng Q. (2020). Crystal plane dependent electrocatalytic performance of NiS_2_ nanocrystals for hydrogen evolution reaction. J. Catal. 381, 63–69. 10.1016/j.jcat.2019.10.038

[B16] Liu T. Ma X. Liu D. Hao S. Du G. Ma Y. (2017). Mn doping of CoP nanosheets array: an efficient electrocatalyst for hydrogen evolution reaction with enhanced activity at all pH values. ACS Catal. 7, 98–102. 10.1021/acscatal.6b02849

[B17] Liu Y. Huang B. Hu X. Xie Z. (2019). Surfactant-assisted hydrothermal synthesis of nitrogen doped Mo_2_C@C composites as highly efficient electrocatalysts for hydrogen evolution reaction. Int. J. Hydrog. Energ. 44, 3702–3710. 10.1016/j.ijhydene.2018.12.096

[B18] Mohammed-Ibrahim J. Sun X. (2019). Recent progress on earth abundant electrocatalysts for hydrogen evolution reaction (HER) in alkaline medium to achieve efficient water splitting – A review. J. Energ. Chem. 34, 111–160. 10.1016/j.jechem.2018.09.016

[B19] Ojha K. Saha S. Dagar P. Ganguli A. K. (2018). Nanocatalysts for hydrogen evolution reactions. Phys. Chem. Chem. Phys. 20, 6777–6799. 10.1039/c7cp06316d 29460931

[B20] Popczun E. J. McKone J. R. Read C. G. Biacchi A. J. Wiltrout A. M. Lewis N. S.. (2013). Nanostructured nickel phosphide as an electrocatalyst for the hydrogen evolution reaction. J. Am. Chem. Soc. 135, 9267–9270. 10.1021/ja403440e 23763295

[B21] Popczun E. J. Read C. G. Roske C. W. Lewis N. S. Schaak R. E. (2014). Highly active electrocatalysis of the hydrogen evolution reaction by cobalt phosphide nanoparticles. Angew. Chem. Int. Ed. 53, 5427–5430. 10.1002/anie.201402646 24729482

[B22] Qu Y. Medina H. Wang S.-W. Wang Y.-C. Chen C.-W. Su T.-Y.. (2016). Wafer scale phase-engineered 1T- and 2H-MoSe_2_/Mo core–shell 3D-hierarchical nanostructures toward efficient electrocatalytic hydrogen evolution reaction. Adv. Mater. 28, 9831–9838. 10.1002/adma.201602697 27717140

[B23] Sohn H. Ozkan U. S. (2016). Cobalt-based catalysts for ethanol steam reforming: an overview. Energy Fuels 30, 5309–5322. 10.1021/acs.energyfuels.6b00577

[B24] Sun S. Li H. Xu Z. J. (2018). Impact of surface area in evaluation of catalyst activity. Joule 2, 1024–1027. 10.1016/j.joule.2018.05.003

[B25] Tan P. Gao Z. Shen C. Du Y. Li X. Huang W. (2014). Ni-Mg-Al solid basic layered double oxide catalysts prepared using surfactant-assisted coprecipitation method for CO_2_ reforming of CH_4_. Chinese J. Catal. 35, 1955–1971. 10.1016/S1872-2067(14)60171-6

[B26] Toloman D. Pana O. Stefan M. Popa A. Leostean C. Macavei S.. (2019). Photocatalytic activity of SnO_2_-TiO_2_ composite nanoparticles modified with PVP. J. Colloid Interface Sci. 542, 296–307. 10.1016/j.jcis.2019.02.026 30763897

[B27] van Bommel A. Dahn J. R. (2009). Analysis of the growth mechanism of coprecipitated spherical and dense nickel, manganese, and cobalt-containing hydroxides in the presence of aqueous ammonia. Chem. Mater. 21, 1500–1503. 10.1021/cm803144d

[B28] Wang C. Wang Y. Chen M. Hu J. Yang Z. Zhang H. (2019). Hydrogen production from ethanol steam reforming over Co–Ce/sepiolite catalysts prepared by a surfactant assisted coprecipitation method. Int. J. Hydrog. Energ. 44, 26888–26904. 10.1016/j.ijhydene.2019.08.058

[B29] Wang J. Xu F. Jin H. Chen Y. Wang Y. (2017). Non-noble metal-based carbon composites in hydrogen evolution reaction: fundamentals to applications. Adv. Mater. 29:1605838. 10.1002/adma.201605838 28234409

[B30] Wu Y. Liu X. Han D. Song X. Shi L. Song Y.. (2019). Electron density modulation of NiCo_2_S_4_ nanowires by nitrogen incorporation for highly efficient hydrogen evolution catalysis. Nat. Commun. 9:1425. 10.1038/s41467-018-03858-w 29651037 PMC5897358

[B31] Xu L. Ma L. Zhou X. Liu Z. Luo D. Xu X. (2018). Boosting electrocatalytic activity of ultrathin MoSe_2_/C composites for hydrogen evolution via a surfactant assisted hydrothermal method. Int. J. Hydrog. Energ. 43, 15749–15761. 10.1016/j.ijhydene.2018.07.041

[B32] Zhang W. Li W. Li Y. Peng S. Xu Z. (2019). One-step synthesis of nickel oxide/nickel carbide/graphene composite for efficient dye-sensitized photocatalytic H_2_ evolution. Catal. Today 335, 326–332. 10.1016/j.cattod.2018.12.016

[B33] Zhao Y. Chang C. Teng F. Zhao Y. Chen G. Shi R. (2017). Defect-engineered ultrathin δ-MnO_2_ nanosheet arrays as bifunctional electrodes for efficient overall water splitting. Adv. Energ. Mater. 7:1700005 10.1002/aenm.201700005

[B34] Zhou H. Yu F. Huang Y. Sun J. Zhu Z. Nielsen R. J.. (2016). Efficient hydrogen evolution by ternary molybdenum sulfoselenide particles on self-standing porous nickel diselenide foam. Nat. Commun. 7:12765. 10.1038/ncomms12765 27633712 PMC5028416

[B35] Zhou W. Jin J. Jia L. Yang L. Chen S. (2016). Recent developments of carbon-based electrocatalysts for hydrogen evolution reaction. Nano Energy 28, 29–43. 10.1016/j.nanoen.2016.08.027

[B36] Zou X. Zhang Y. (2015). Noble metal-free hydrogen evolution catalysts for water splitting. Chem. Soc. Rev. 44, 5148–5180. 10.1039/c4cs00448e 25886650

