# Supplementary material for: Effect of PEG on Performance of NiMnO Catalyst for Hydrogen Evolution Reaction
Source: Front Chem. 2020 Apr 23;8:281. doi: 10.3389/fchem.2020.00281 (PMC7191312; doi:10.3389/fchem.2020.00281)
Supplement: Supplementary file 1 [file Data_Sheet_1.PDF]

Supporting information for

Effect of PEG on performance of NiMnO catalyst for hydrogen evolution reaction

*Jinfeng Zeng[a], Lu Chen[b], Linlin Li[a], Wei Yang[*a], Hanbo Zou[a], Shengzhou Chen[* c]*

[a] School of Chemistry and Chemical Engineering, Guangzhou University, Guangzhou 510006, P.R. China.

[b] School of Biotechnology and Health Science, Wuyi University, Jiangmen, Guangdong 529090, China.

[c] Guangzhou Key Laboratory for New Energy and Green Catalysis, Guangzhou University, Guangzhou, 510006, China.

* Corresponding author. E-mail: wyang@gzhu.edu.cn; szchen@gzhu.edu.cn

Tel: +86-13719030050

Figure s1 EDS of NiMnO catalysts.

Spectrum processing:

Peaks omitted: 2.062, 2.328 keV

Processing option: All elements analyzed (Normalized)

Number of iterations = 3

Standard:

C	FeS2C	19-Oct-2012 04:44 PM
O	SiO2	1-Jun-1999 12:00 AM
Mn	Mn	1-Jun-1999 12:00 AM
Ni	Ni	1-Jun-1999 12:00 AM

Element	Weight %	Atomic %
C K	0.37	0.76
O K	48.43	75.97
Mn K	46.88	21.42
Ni K	4.33	1.85
Total	100.00	

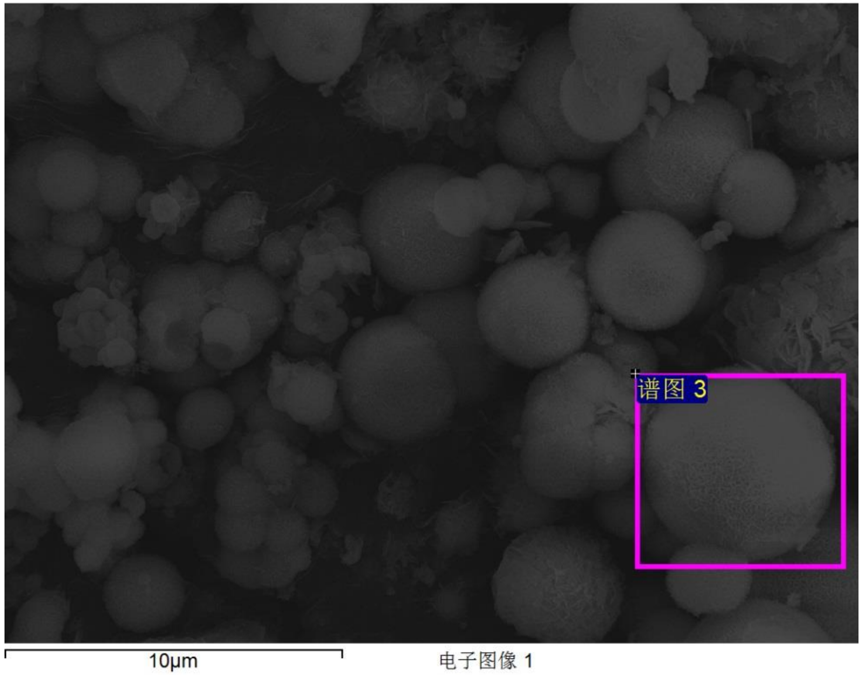

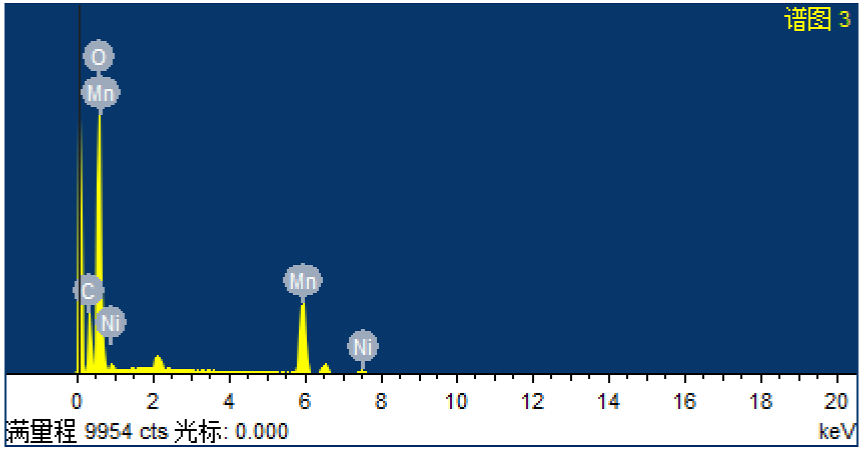

Note： NMO

Spectrum processing:

Peaks omitted: 2.062, 2.320, 9.410 keV

Processing option: All elements analyzed (Normalized)

Number of iterations = 3

Standard:

C FeS2C 19-Oct-2012 04:44 PM

O SiO2 1-Jun-1999 12:00 AM

Mn Mn 1-Jun-1999 12:00 AM

Ni Ni 1-Jun-1999 12:00 AM

Element	Weight %	Atomics %
C K	0.30	0.73
O K	37.38	66.94
Mn K	55.03	28.72
Ni K	4.99	2.43
Total	100.00	

Note： NMO-PEG-2g

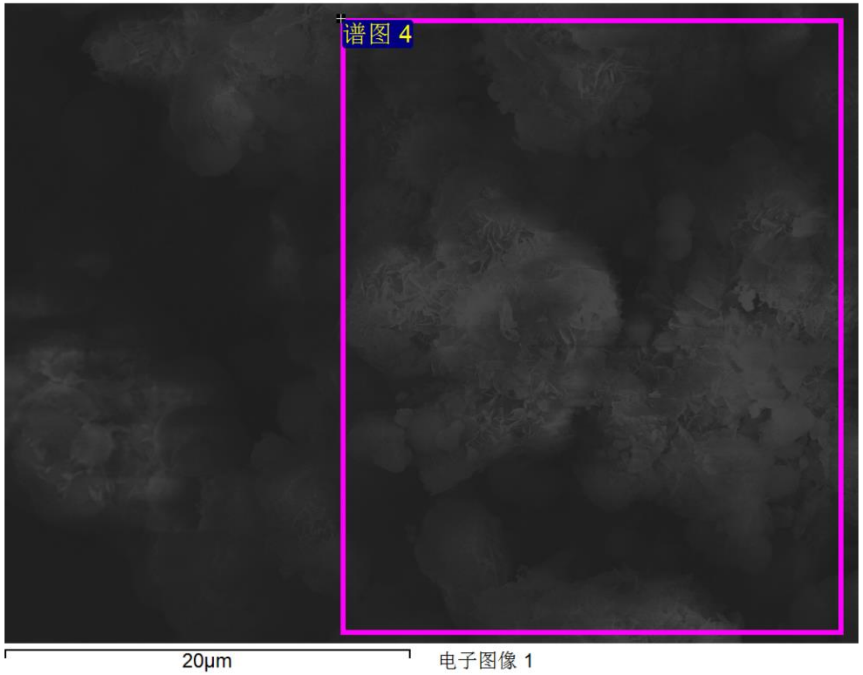

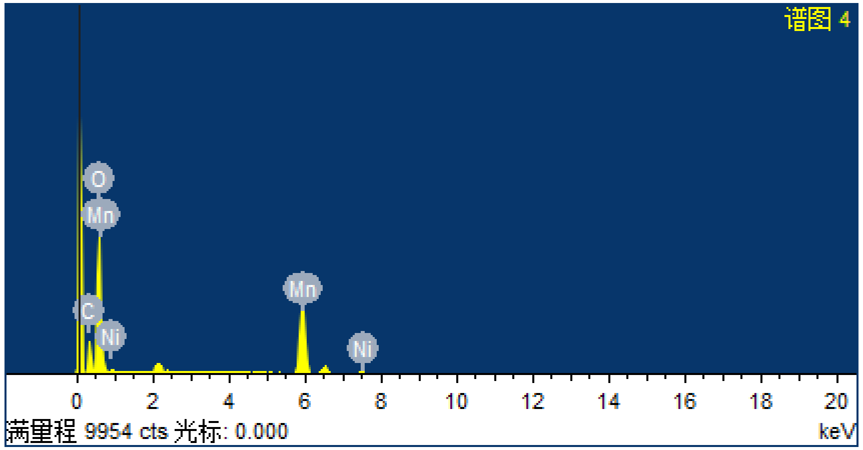

OXFORD INSTRUMENTS
The Business of Science

Spectrum processing:

Peaks omitted: 2.055 keV

Processing option: All elements analyzed (Normalized)

Number of iterations = 3

Standard:

C FeS2C 19-Oct-2012 04:44 PM

O SiO2 1-Jun-1999 12:00 AM

Mn Mn 1-Jun-1999 12:00 AM

Ni Ni 1-Jun-1999 12:00 AM

Element	Weight %	Atomics %
C K	0.48	0.99
O K	50.24	77.11
Mn K	45.20	20.20
Ni K	4.07	1.70
Total	100.00	

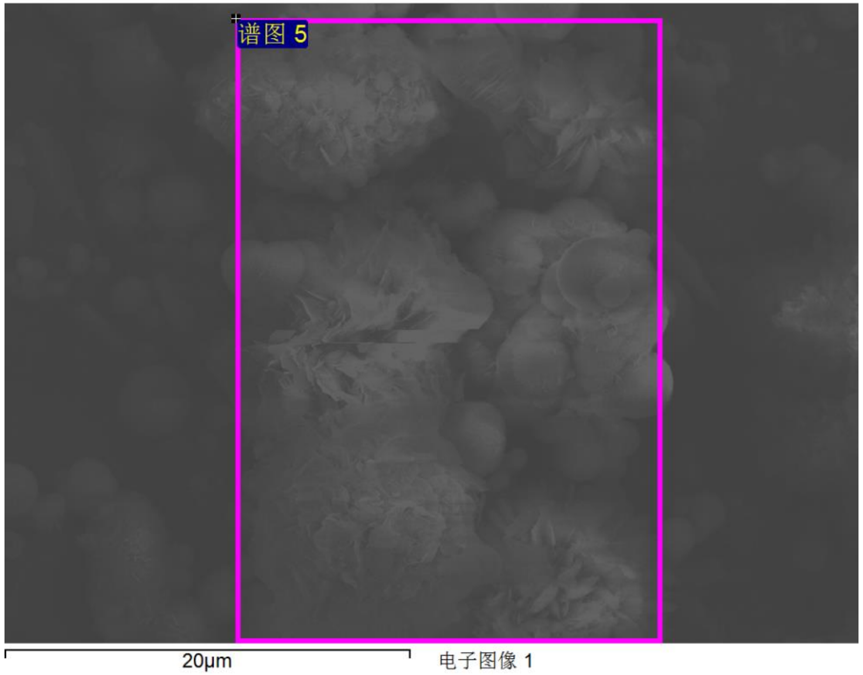

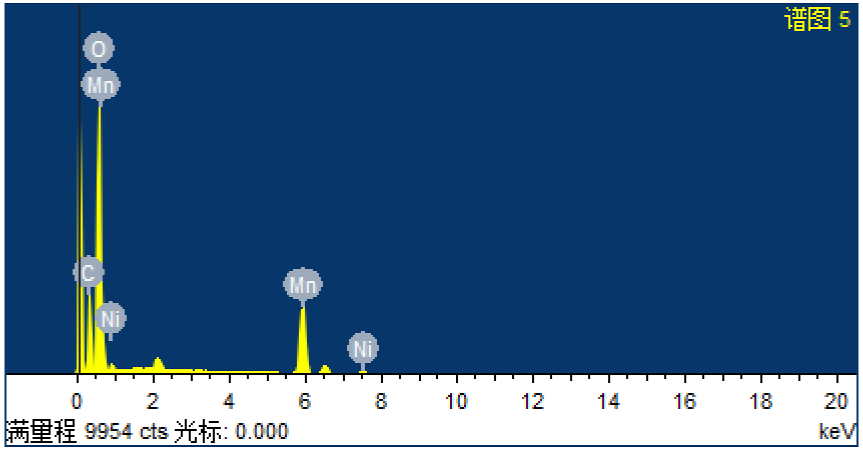

Note：NMO-PEG-3g

OXFORD INSTRUMENTS The Business of Science

Figure s2 Nitrogen adsorption–desorption isotherms and pore size distribution of NiMnO catalysts.

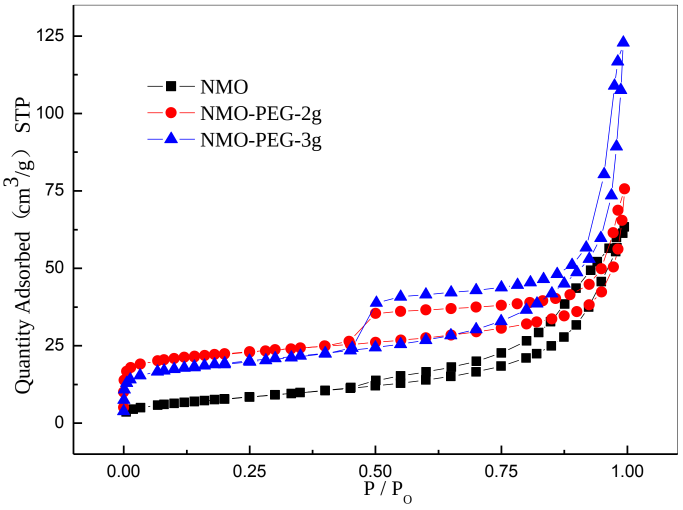

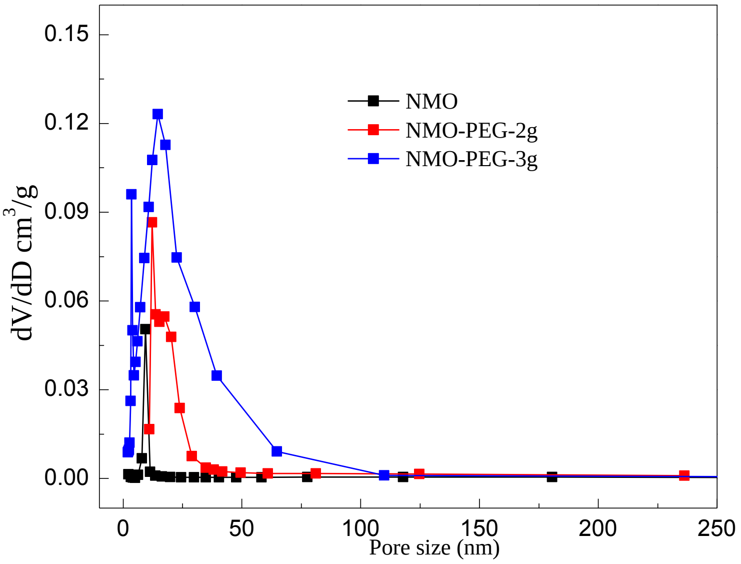

Table. s1 Element ratio value from ICP-AES.

sample	Element	Weight %	Atomic %
NMO	Mn	91.55	92.05
	Ni	8.45	7.95
NMO-PEG-2g	Mn	90.75	91.29
	Ni	9.25	8.71
NMO-PEG-3g	Mn	91.62	92.21
	Ni	8.38	7.89
